# Development and validation of children’s stories as a health education strategy in speech, language, and hearing sciences

**DOI:** 10.1590/2317-1782/20212021309en

**Published:** 2022-08-19

**Authors:** Laura Lima Costa, Tatiane Martins Jorge

**Affiliations:** 1 Faculdade de Medicina de Ribeirão Preto, Universidade de São Paulo – USP - Ribeirão Preto (SP), Brasil.; 2 Departamento de Ciências da Saúde, Faculdade de Medicina de Ribeirão Preto, Universidade de São Paulo – USP - Ribeirão Preto (SP), Brasil.

**Keywords:** Juvenile Literature, Speech, Language and Hearing Sciences, Child, Health Education, Self-care

## Abstract

**Purpose:**

Developing and validating children’s stories focused on health education in Speech, Language, and Hearing Sciences.

**Methods:**

Cross-sectional, descriptive, quali-quantitative two-step study. 1st) elaboration of 10 children’s stories, guiding questions, explanatory notes, and illustrations. 2nd) validation by judges (speech therapists, educators, and familiar) in two phases. In the first, the sample consisted of 42 judges and in the second, 28. To access the materials and the validation questionnaire, a website was made available. The questionnaire investigated the judges’ personal data and the judges’ perceptions regarding the contents, vocabulary, illustrations, structure, and motivation of the materials. A five-point Likert scale of the agreement was used, with spaces for suggestions in each question. The quantitative analysis was done using the Content Validity Index and the Content Validity Coefficient. The qualitative analysis was based on the comments of the judges. The minimum rate of agreement among the judges on the decision to change or not change the materials was 80%.

**Results:**

The first phase pointed out the need for modifications in three titles and two vocabularies (bullying and dyke), and the need for increasing to five years of age the minimum age for access to the materials. The second phase revealed good acceptability in all changes made.

**Conclusion:**

The materials developed were validated and can be used as health education tools by speech therapists and educators, as well as parents.

## INTRODUCTION

In health education, children’s stories can be used as prevention strategies, teaching children to choose healthy habits^(1)^. They are great allies in the development of imagination, child care, communication, and bonds between adults and children^(2)^. In addition, children’s stories allow the child to get involved in the theme, observing the events that surround the characters, which can also be part of their daily life, which strengthens cognitive, social, and emotional development^(3)^. Other benefits of children’s storytelling are described in the literature: favoring the understanding of narrative texts when reading is dialogic^(4)^ and vocabulary expansion in the presence of an adult who directs the reading from guiding questions^(5)^. The illustrations, when present, associate non-verbal and verbal elements of the narrative, synthesizing events^(6)^.

In Speech-Language Pathology, although there are booklets^(7)^ and websites^(8)^ on child development, no validated children’s stories about child self-care related to health education in Speech-Language Pathology and Audiology were found.

The construction of stories or other educational materials must involve validation steps, due to the importance of disseminating reliable information to the population, providing educational and motivating actions in health education for transformations^(9)^.

Validation by judges increases the reliability of the instrument^(10)^. Recent research has reinforced the importance of the same material being evaluated by different categories of judges, such as specialists in the studied area, family members, and early childhood education professionals^(7)^.

For validation, the judges analyze different aspects of the material under construction, such as content, form, vocabulary, motivation, and relevance to the presented theme, and may also make comments, suggestions, and criticisms^(7,11,12)^. After being validated, the material can be used in different ways and scenarios, but always focused on health education.

Thus, considering that no research was found in the national literature on the validation of children’s stories that encourage self-care in the field of Speech Therapy, this study was designed to develop and validate a series of children’s stories aimed at health education in Speech-Language Pathology and Audiology.

## METHODS

This is a cross-sectional, descriptive, qualitative-quantitative study, structured in two stages, and approved by the Research Ethics Committee of the Hospital das Clínicas, Faculty of Medicine of Ribeirão Preto, University of São Paulo (CAAE: 39492120.9 .0000.5440 and Protocol Number: 4,445,712). All participants consented to participation, after reading the Informed Consent Form (ICF) online and declaration of acceptance to participate.

The first stage involved the elaboration of educational materials and the second involved the validation of the materials.

### Preparation of educational materials

**Figure 1:**
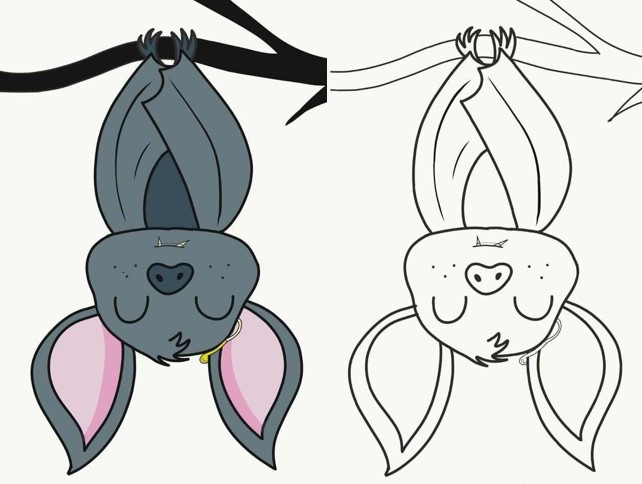
Character “Little Bat” in color and black and white versions

Ten children’s stories were created for children from four to 10 years old, about some situations that can happen during child development, in the context of communication and food. The context of the stories was a kingdom with anthropomorphic animals that lived in society. The kingdom was called “The Kingdom of Rabbits”. The main characters were illustrated with color, being also available in black and white to be printed and colored by the children. An example of the illustrations can be seen in Figure 1. For each story, explanatory notes were prepared for family members and caregivers who deepen the themes portrayed in the stories and guiding questions for children. In Chart 1, the title, theme, and synopsis of each story are presented.

**Chart 1:** Titles of children’s stories, themes portrayed, and synopses

Story titles	Themes	Synopses
The Bunny Princess and the Scream	Abusive vocal behavior, focusing on screaming	Presentation of the Kingdom of Rabbits, of Princess Bunny who communicated only by screaming, and how the Fairy made her understand that screaming harmed her relationship with her interlocutors and caused changes in her voice.
The Messenger Pigeon	Reading and writing changes	A baby pigeon had difficulties in oral and written language. His dream was to be a real messenger like his parents, but he couldn’t due to difficulties. The Fairy intervened, teaching him to speak and write well, so she was able to fulfill her dream.
The Good Ear	Unilateral hearing loss and the importance of auditory rehabilitation.	The Fairy was watching a baby bat with difficulty hearing in one ear and the ability to locate sound, which made it impossible to fly. Recognizing the presence of a hearing loss, the Rabbit Queen ordered a hearing aid. The puppy adapted to the device and was able to fly thanks to the intervention.
The Health System	Implementation of a free and universal Health System.	The public health system was created by the rulers of the Kingdom of Rabbits, and there was a public presentation of the Fairy. It was clarified that problems could be solved without using magic.
The Swimming Duck	Swimmer’s ear and consequences for speech and hearing.	One of the ducklings had earache after swimming and speech disorders as a result of recurrent ear infections. A way to prevent earache was presented, and with that language could be developed without difficulties.
The Castor and the Pacifier	Pacifier use and impact for orofacial development.	Dental alterations were portrayed by the use of the pacifier, which prevented the chick from using its teeth to gnaw wood, an essential activity for beavers.
Chico and Food	Importance of food variety for the development of the stomatognathic system	The importance of food variation, different consistencies, hardness, and types of food, for the development and strengthening of the stomatognathic system, was portrayed.
Lulu and the stuffy nose	Mouth breathing and impact on orofacial and body development.	The oral breather was portrayed due to an allergic condition. The objective was to show anatomical changes, stomatognathic, emotional, and cognitive functions that this type of breathing can cause in child development.
Kiki and the Stutter	Developmental stuttering and bullying.	A pup named Eduardo was nicknamed Kiki and was bullied at school for having a developmental stutter. In the family environment, it was not much different. Even so, he felt motivated to achieve his goal, to be a reporter, like his father.
The Fairy’s Profession	Role of the Speech-Language Pathologist. Importance of the creation of Speech-Language Pathology Science	The Fairy’s performance became known in other realms, and during an event for fraternization in the Kingdom of Rabbits, the appointment of the Fairy’s profession was discussed; and the name chosen was Fonoaudiologia.

### Sample: invitation and access to materials

The judges were invited through the researchers’ social networks. The invitation provided instructions on the ICF and on access to the website that contained the educational materials.

The website was created exclusively to disseminate the research, for the group of judges to access the materials, and was composed of the following pages: Home (which described the study proposal); Children’s Stories (with illustrations and guiding questions); Explanatory Notes; Who we are (with information about the researchers, the illustrator and ways to get in touch). There was also an emphasis on the online questionnaire that should be answered by the study judges after reading and checking the entire website.

To participate, the judges had to meet the following inclusion criteria: being a Speech-Language Pathologist from public and/or private services, with a minimum time of work of two years, regardless of the area of ​​work; be an early childhood education educator and/or the first cycle of elementary education in public and/or private schools, with a minimum of five years of experience; be a family member of children between four and 10 years old. The judges’ gender and federative unit were not considered inclusion criteria. In the 1st validation phase, which took place between March and May 2021, 42 judges participated: 10 Speech-Language Pathologists, 18 educators, and 14 family members. In the 2nd phase, between August and October of the same year, 28 responded to the new questionnaire, including nine Speech-Language Pathologists, nine educators, and 10 family members. The definition of the sample number by groups followed the recommendation of Lynn^(13)^, according to which a minimum of six judges already ensures the validation of the analyzed content. The individuals excluded from the sample were those who agreed to participate but did not respond to the questionnaire.

### Collection instrument

The questionnaire contained two parts: the first investigated the judges’ personal and/or professional data (five questions) and the second inquired about the materials developed: content, vocabulary, illustration, structure, and motivation (20 questions). The questions that made up the investigated domains can be seen in Chart 2.

**Chart 2:** Domains and their equivalent questions about the materials evaluated by the judges in the 1st validation phase

Domains	Nº	Questions
Content	Q1	The content of the stories is suitable for children.
	Q2	Educational information relevant to the target audience was presented.
	Q3	The sequence of children’s stories is logical and coherent.
	Q4	Stories are important for children’s self-care.
	Q5	The stories can circulate in the scientific environment of the area.
	Q6	The information contained in the stories can be considered a health education strategy.
	Q7	The guiding questions for children’s understanding of the content are coherent.
	Q8	The titles of the stories are pertinent.
	Q9	Explanatory notes for family members, educators, or caregivers are important.
	Q10	Explanatory notes for family members, educators, or caregivers are clear.
Vocabulary	Q11	Vocabulary is accessible to children.
	Q12	The texts (of the stories and explanatory notes) are clear and objective.
Illustration	Q13	The images are easily recognized by children.
	Q14	The images arouse children’s interest in the stories.
Structure	Q15	Text formatting, font size, and font are adequate.
	Q16	The organization of the text and illustrations is adequate.
Motivation	Q17	The content is a motivator for behavior change.
	Q18	The content sparks interest.
	Q19	The materials can be used by any caregiver.
	Q20	The materials propose the construction of knowledge for children and their caregivers.

The judges should respond based on a five-point Likert scale of agreement, ranging from 1 to 5, with 1 = strongly disagree; 2= somewhat disagree; 3= neither agree nor disagree; 4= somewhat agree; 5= strongly agree, as recommended by recent literature^(7,12)^. The judges could also choose the option “don’t know or don’t want to answer”.

For all questions, there was a blank space for the judge to comment on the items, in order to offer suggestions for improving the materials. The estimated time for completing the questionnaire was 30 minutes.

The Checklist for Reporting Results of Internet E-Surveys (CHERRIES)^(14)^ guideline was used because it is recommended in electronic surveys that involve online questionnaires to minimize possible errors.

### Data analysis

Data were organized in a Microsoft® Excel® spreadsheet, 2016. For the descriptive analysis of quantitative variables, mean, median, and standard deviation were calculated. Categorical variables were described by frequency and percentage.

Content validity was assessed by quantitative and qualitative measures. The qualitative analysis was based on the comments made by the judges. Regarding the quantitative analysis, the Content Validity Index (CVI) and the Content Validity Coefficient (CVC) were calculated to verify the degree of agreement of the judges.

The CVI was calculated by dividing the number of judges who partially and strongly agreed with each item investigated by the total number of judges. The calculation was made for each item, for each domain, and considering each group of judges separately and overall (all judges). The decision to use full and partial agreement was based on the study by Alexandre and Coluci^(15)^. The minimum rate of agreement between the judges on the decision to change or not the stories, as well as the explanatory notes and the guiding questions was 80%, as recommended by Lynn^(13)^ and Yusoff^(16)^.

The CVC was calculated according to the judges’ answers, in five steps:

1. The average response of each investigated item was calculated; 2) each means was divided by the maximum possible value of each response (five), resulting in CVCi; 3) the error (Pei) was calculated to discount possible biases of the judges for each question; 4) CVCi was subtracted from Pei, resulting in CVCc for each question; 5) the total CVC (CVCt) was calculated from the average of all questions for each of the domains. Values ​​lower than 0.8 indicated the need for revision^(17)^.

## RESULTS

Regarding the characterization of the sample, with regard to Speech-Language Pathologists, the age ranged from 24 to 45 years (mean 33.2 years, median 31 years, and standard deviation 8.7 years). The time of work ranged from two to 20 years (mean 9.8 years, median 7 years, and standard deviation 7.7 years). Most worked in language (90%) and orofacial motricity (60%). The others had experience in other areas such as fluency (40%), educational speech therapy (30%), audiology (20%), voice (20%), and dysphagia (20%).

The educators who participated as judges were aged between 27 and 58 years (mean 43.1 years, median 43 years, standard deviation 8.3 years). The professional experience ranged from five to 33 years (mean 16.5 years, median 16.5 years, standard deviation 7.3 years). Regarding the level of education, 61% worked in early childhood education, 50% in elementary school, and 10% in early childhood education centers.

As for family members, age ranged between 24 and 51 years (mean 37.3 years, median 36.5 years, standard deviation 8.1 years). With regard to the level of education, 43% completed higher education, 29% secondary education, 14% elementary school, and 14% did not complete elementary school.

The CVI (by groups of judges and general) for each of the investigated domains (content, vocabulary, illustrations, structure, motivation) of the first phase can be seen in Table 1.

**Table 1:** Content Validity Index (by categories of judges and general) in relation to the domain “content” and “vocabulary” of the elaborated materials

Domains	Judges’ response on a 5-point Likert scale and CVI calculation (%)									
	Speech-Language Pathologists (10)			Educators (18)			Family members (14)			General
CONTENT	1, 2 or 3	4 or 5	CVI	1,2 or 3	4 or 5	CVI	1,2 or 3	4 or 5	CVI	CVI
The content of the stories is suitable for children.	0	10	100	0	18	100	0	14	100	100
Relevant educational information was presented to the public.	0	10	100	0	18	100	0	14	100	100
The sequence of children’s stories is logical and coherent.	0	10	100	0	18	100	0	14	100	100
Stories are important for children’s self-care.	0	10	100	0	18	100	0	14	100	100
The stories can circulate in the scientific environment of the area.	0	10	100	0	18	100	0	14	100	100
The information contained in the stories can be considered as a health education strategy.	0	10	100	0	18	100	0	14	100	100
The guiding questions for understanding the content for children are coherent.	0	10	100	0	18	100	0	14	100	100
The titles of the stories are pertinent.	0	10	100	0	18	100	3	11	**78.5** *	92
Explanatory notes for family members, educators or caregivers are important.	0	10	100	0	18	100	0	13	92	97.33
Explanatory notes for family members, educators or caregivers are clear.	0	10	100	0	18	100	0	13	92	100
Domain Total			100			100			96	98.93
VOCABULARY	1. 2 or 3	4 or 5	CVI	1.2 or 3	4 or 5	CVI	1.2 or 3	4 or 5	CVI	CVI
Vocabulary is accessible to children.	1	9	90	0	18	100	2	12	85	91.66
The texts (of the stories and explanatory notes) are clear and objective.	1	9	90	0	18	100	0	14	100	96.66
Domain Total			90			100			92.5	94
ILLUSTRATION	1. 2 or 3	4 or 5	CVI	1.2 or 3	4 or 5	CVI	1.2 or 3	4 or 5	CVI	CVI
The images are easily recognized by children.	0	10	100	0	18	100	0	14	100	100
The images arouse children’s interest in the stories.	0	10	100	1	17	94	1	13	92	95.33
Domain Total			100			97			96	97.66
STRUCTURE	1. 2 or 3	4 or 5	CVI	1.2 or 3	4 or 5	CVI	1.2 or 3	4 or 5	CVI	CVI
Text formatting, font size, and font are adequate.	1	9	90	1	17	94	0	13	92	92
The organization of the text and illustrations is adequate.	1	9	90	1	17	94	1	13	92	92
Domain Total			90			94			92	92
MOTIVATION	1. 2 or 3	4 or 5	CVI	1.2 or 3	4 or 5	CVI	1.2 or 3	4 or 5	CVI	CVI
The content is a motivator for behavior change.	0	10	100	0	18	100	0	13	92	97.33
The content sparks interest.	1	9	90	0	18	100	0	14	100	96.66
The materials can be used by any caregiver.	1	9	90	0	18	100	1	13	92	92
The materials propose the construction of knowledge for children and their caregivers.	0	10	100	0	18	100	0	14	100	100
Domain Total			95			100			96	97
General CVI			96			98.2			94.5	95.92

* Value lower than the minimum agreement of 80%

Many positive comments were made by the judges: “The stories contextualize the children’s playful universe”; “The sequence of stories gives the impression of continuity as if the kingdom were real”; “Very instructive and captivating”, “They help to work and understand the daily situations of the profession”; “My seven-year-old son completed the learning by himself at the end of each story”.

Only the “title” aspect of the “content” domain had a CVI of less than 80% for the family group, while the CVC was borderline at 0.81. The comment made by a speech-language pathologist judge helped to understand the low CVI, allowing changes in the titles of three stories:

*“All titles address the main character, except the bat story (‘The Good Ear’). In the story ‘The Bunny Princess and the Scream’ I would change the sentence structure to ‘The Princess Scream’, and I also did not find the title ‘Health System’ interesting/inviting for children”.*

Although all aspects of the “vocabulary” domain presented CVI above 80%, the researchers made changes to some words in the story based on the comments made by two judges (family and Speech-Language Pathologist, respectively): “give an example about what is bullying”, “dam is a word that is difficult to understand depending on age”.

Regarding the “illustration” and “structure” domains, the CVI was higher than 80% and the judges’ comments were positive: “sufficient formatting”, “it is clear”, “perfect organization”.

Although the CVI for the “Motivation” domain was above 80%, a comment made by the family group drew the researchers’ attention to necessary changes: “perhaps for older children, aged nine or 10, the content is not enough to motivate them by the fact that it is quite childish”, “I thought the stories are a little long, depending on the age”.

**Figure 2:**
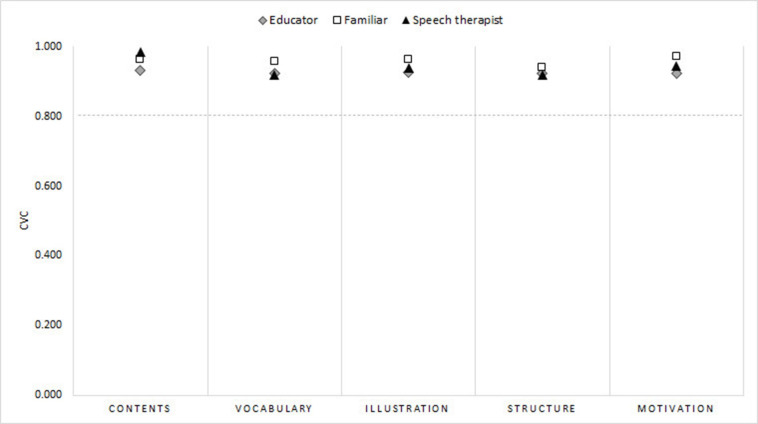
CVCt values of the investigated domains for each judging category in the first validation phase

The CVC values ​​of each investigated item ranged from 0.81 to 1.0. As for the total CVC of the domains, according to the groups of judges (Figure 2), the values ​​ranged from 0.91 to 0.98, as shown below: for “content”, the CVCt of the educators was 0 .93, family members 0.96, and Speech-Language Pathologists 0.98, respectively for the remaining domains “vocabulary”, 0.92, 0.95 and 0.91; “illustration”, 0.92, 0.96 and 0.93; “structure”, 0.92, 0.94 and 0.91; and “motivation”, 0.92, 0.97, and 0.94. The overall CVC was: family members, 0.96; educators, 0.92; and Speech-Language Pathologists, 0.94.

After the qualitative analysis of the judges, there were changes in three main aspects: 1) title of the stories, with the title “The Bunny Princess and the Scream” being replaced by “The Princess Bunny’s Scream”, “The Good Ear” by “The Ear of the Bat” and “The Health System” for “Health for All”; 2) vocabulary: the term bullying was explained and the word “dam” was replaced by “barrage”; 3) Minimum recommended age: increased from four to five years.

In the second validation phase, after the changes were made according to the qualitative and quantitative analyses, the judges answered the new questionnaire composed of: “The new vocabulary is accessible to children” and “The new titles of the stories are relevant”. CVI can be seen in Table 2.

**Table 2:** Content Validity Index of the 2nd phase of validation of the elaborated materials

Questions	Judges’ response on a 5-point Likert scale and CVI calculation (%)									
	Speech-Language Pathologists (9)			Educators (9)			Family members (10)			General
	1, 2 or 3	4 or 5	CVI	1,2 or 3	4 or 5	CVI	1,2 or 3	4 or 5	CVI	CVI
The new vocabulary is accessible to children	0	9	100	1	8	88.88	2	8	80	89.62
The new story titles are pertinent.	0	9	100	1	8	88.88	0	10	100	96.29
Total			100			88.88			90	92.95

Vocabulary and title CVC for family members were 0.97 and 0.96; respectively; for educators, 0.88 and 0.9; and Speech-Language Pathologists, 0.97 in both.

## DISCUSSION

The process of validating the educational materials involved different perspectives on the same object^(7)^, as well as different analyzes for their improvement.

Regarding family members, most had an education level between complete high school and incomplete elementary school, which has also been mentioned by the Brazilian census. According to the Brazilian Institute of Geography and Statistics (2020), in people aged 25 years or older, the minority has completed higher education (17.4%)^(18)^.

The diversity of the family’s level of education was favorable, as it allowed the materials to be judged by people from different backgrounds and social scenarios. There were no reports of difficulty in understanding the content of the stories due to the vocabulary used, or in understanding the proposal of the materials, which suggests that they can be used by family members regardless of the level of instruction.

Likewise, the variety of Speech-Language Pathologists in terms of specialties and educators in terms of teaching levels was important for judging the materials.

The quantitative results, according to the CVI and CVC of the first phase, indicated a high acceptability value, indicating only the need for revision in the titles of the stories. The qualitative analysis, based on the judges’ comments, revealed other aspects to be improved, which indicates the importance of integrating the two types of analysis.

Regarding the five domains analyzed, “content”, “vocabulary” and “motivation” were the ones that received suggestions for improvement by the judges. According to the literature^(19)^, content and vocabulary are the domains that most have changed in the validation of educational materials.

Modifying vocabulary implies excluding, adding, and/or replacing words and reformulating sentences^(19)^. In the present study, the vocabulary was adapted to the children’s audience in the stories, aiming at facilitating the reader’s understanding of the content^(20)^. Although the word “dam” could favor the expansion of vocabulary as it is not a common word, the change to “dam” aimed to facilitate the explanation and understanding of the construction process carried out by the beavers, characters in one of the stories. The exemplification of behaviors linked to bullying, such as laughing and calling names, was also important to expand the child’s understanding of this type of violence.

In the “content” domain, about the content of the stories, there was good acceptance by the judges, with only changes in the title of three stories.

The guiding questions for understanding the content were considered coherent by the judges. Telling stories with the mediation of guiding questions allows checking the content learned, in addition to increasing the complexity of the narrative discourse^(21)^.

Regarding the “motivation” domain, the materials developed were considered capable of arousing interest, generating knowledge in children and caregivers, and changing behaviors. However, as suggested by the judges, it was considered important to change the age group of the target audience of the stories. The minimum age has been increased to five years, and it will be recommended that up to seven years the counting be intermediated by adults, as recommended by the Ministry of Health^(22)^. Children over seven years old, who have already acquired a written code of the Portuguese language, should be encouraged to read alone so that their autonomy and decision-making are enhanced^(23)^.

The children’s story illustrations were well accepted by the judges. The use of this visual resource captures the reader’s attention^(6,24)^, and may focus on some specific elements that contribute to learning^(24)^.

The changes made it possible to increase the quality of the materials produced^(25)^, in addition to showing the commitment of each contributing judge^(26)^. The specificity of the judges’ notes facilitated the process of rewriting the materials. The second validation phase, performed after the modifications, also indicated values ​​above the minimum agreement of 80% for CVI and CVC. The items “vocabulary” and “titles” were again appreciated and well accepted by the judges.

The importance of the materials was perceived by the judges, according to the quantitative results and qualitative analysis. These judges recognized that children’s stories are good health education strategies, which can favor the development of children’s self-care^(26)^, in addition to promoting the importance of speech therapy. Speech-Language Pathologists, family members, and education professionals will be able to use this tool to discuss issues about child health with students and their caregivers.

The limitations of the study were related to the decrease in the number of judges between the first and second phases of validation of the materials, from 42 to 28 judges. Although existing, this decrease did not influence the minimum number of judges needed for the validation process. One of the possible causes could have been the time gap between the validation phases.

It is expected that the elaborated and validated materials will be published in book format, digital and physical, and disseminated to Speech-Language Pathologists, educators, and children’s families aiming at health education and reading stimulation.

## CONCLUSION

The elaborated materials had good acceptability by the group of judges. The validation process reinforced the importance of combining different people and analyses in improving educational tools.

The judges’ comments were fundamental to pointing out the necessary changes: three titles were modified, technical terms were explained and/or replaced, and the suggested minimum age for access to the materials was increased to five years.

After the modifications that were made, it is expected that the materials can be used in the routine of the Speech-Language Pathology and Education professionals, and can arouse the interest of children and family members in Speech-Language Pathology and Audiology in child development, favoring reflections on self-care in childhood.
