# Supplementary material for: Development and validation of children’s stories as a health education strategy in speech, language, and hearing sciences
Source: Codas. 2022 Aug 19;34(6):e20210309. doi: 10.1590/2317-1782/20212021309en (PMC9886290; doi:10.1590/2317-1782/20212021309en)
Supplement: Translation

# Translation: Elaboração e validação de histórias infantis como estratégia de educação em saúde na fonoaudiologia

**DOI:** 10.1590/2317-1782/20212021309pt

**Authors:** Laura Lima Costa, Tatiane Martins Jorge

**Affiliations:** 1 Faculdade de Medicina de Ribeirão Preto, Universidade de São Paulo – USP - Ribeirão Preto (SP), Brasil.; 2 Departamento de Ciências da Saúde, Faculdade de Medicina de Ribeirão Preto, Universidade de São Paulo – USP - Ribeirão Preto (SP), Brasil.

**Keywords:** Literatura Infantil, Fonoaudiologia, Criança, Educação em Saúde, Autocuidado

## Abstract

**Objetivo:**

Desenvolver e validar histórias infantis voltadas para a educação em saúde na Fonoaudiologia.

**Método:**

Estudo transversal, descritivo, quali-quantitativo de duas etapas. 1ª) elaboração de 10 histórias infantis, questões norteadoras, notas explicativas e ilustrações. 2ª) validação por juízes (fonoaudiólogos, educadores e familiares) em duas fases. Na primeira, a amostra foi composta por 42 juízes e na segunda por 28. Para acessar os materiais e o questionário de validação, foi disponibilizado um website. O questionário investigava os dados pessoais dos juízes e a percepção dos juízes em relação ao conteúdo, ao vocabulário, às ilustrações, à estrutura e à motivação dos materiais. Utilizou-se escala Likert de concordância de cinco pontos, com espaços para sugestões em cada questão. A análise quantitativa ocorreu por meio do Índice de Validade do Conteúdo e do Coeficiente de Validade de Conteúdo. A análise qualitativa ocorreu a partir dos comentários feitos pelos juízes. O índice mínimo de concordância entre os juízes sobre a decisão de alterar ou não os materiais foi de 80%.

**Resultados:**

A primeira fase apontou necessidade de modificar três títulos e dois vocabulários (*bullying* e dique), e de aumentar a idade mínima sugerida para acesso aos materiais para cinco anos de idade. A segunda fase revelou boa aceitabilidade em todas as mudanças realizadas.

**Conclusão:**

Os materiais elaborados foram validados e podem ser usados como ferramentas de educação em saúde por fonoaudiólogos e educadores, e também pelos familiares.

## INTRODUÇÃO

Na educação em saúde, as histórias infantis podem ser utilizadas como estratégias de prevenção, orientando, de modo pedagógico, crianças a escolherem hábitos saudáveis^(1)^. São ótimas aliadas no desenvolvimento da imaginação, dos cuidados infantis, da comunicação e de vínculos entre adultos e crianças^(2)^. Além disso, as histórias infantis permitem que a criança se envolva na temática, observando os acontecimentos que rodeiam as personagens, que também podem fazer parte do seu cotidiano, o que fortalece o desenvolvimento cognitivo, social e emocional^(3)^. Outros benefícios da contação de histórias infantis são descritos na literatura: favorecimento da compreensão de textos narrativos quando a leitura é dialógica^(4)^ e expansão do vocabulário na presença de um adulto que dirige a leitura a partir de questões norteadoras^(5)^. As ilustrações, quando presentes, associam elementos não verbais aos verbais da narrativa, sintetizando acontecimentos^(6)^.

Na Fonoaudiologia, apesar de existirem cartilhas^(7)^ e websites^(8)^ sobre o desenvolvimento infantil, não foram encontradas histórias infantis validadas sobre o autocuidado infantil relacionado à educação em saúde em Fonoaudiologia.

A construção de histórias ou outros materiais educativos deve envolver etapas de validação, devido a importância de divulgar informações confiáveis à população, proporcionar ações em educação em saúde de caráter educativo e motivador para transformações^(9)^.

A validação por juízes aumenta a confiabilidade do instrumento^(10)^. Pesquisa recente tem reforçado a importância de um mesmo material ser avaliado por diferentes categorias de juízes, como especialistas da área estudada, familiares e profissionais da educação infantil^(7)^.

Para a validação, os juízes analisam diferentes aspectos do material em construção, como conteúdo, forma, vocabulário, motivação e relevância para a temática apresentada, podendo também fazer comentários, sugestões e críticas^(7,11,12)^. Após ser validado, o material pode ser utilizado de diferentes maneiras e cenários, mas sempre voltado à educação em saúde.

Assim, considerando que na literatura nacional não foram encontradas pesquisas sobre a validação de histórias infantis que incentivem o autocuidado na área da Fonoaudiologia, este estudo foi delineado com o objetivo de desenvolver e validar uma série de histórias infantis voltadas para a educação em saúde na Fonoaudiologia.

## MÉTODO

Trata-se de um estudo transversal, descritivo, de caráter quali-quantitativo, estruturado em duas etapas, tendo sido aprovado pelo Comitê de Ética em Pesquisa do Hospital das Clínicas da Faculdade de Medicina de Ribeirão Preto da Universidade de São Paulo (CAAE: 39492120.9.0000.5440 e Número do Parecer: 4.445.712). Todos os participantes consentiram com a participação, após leitura do Termo de Consentimento Livre e Esclarecido (TCLE) online e declaração de aceite em participar.

A primeira etapa contou com a elaboração dos materiais educativos e a segunda envolveu a validação dos materiais.

### Elaboração dos materiais educativos

Foram criadas 10 histórias infantis voltadas para crianças de quatro a 10 anos de idade, sobre algumas situações que podem acontecer durante o desenvolvimento infantil, no âmbito da comunicação e da alimentação. O contexto das histórias foi um reino com animais antropomórficos que viviam em sociedade. O reino foi denominado “O Reino dos Coelhos”. As personagens principais foram ilustradas com cores, sendo disponibilizadas, também, em preto e branco para serem impressas e coloridas pelas crianças. Um exemplo das ilustrações pode ser visualizado na Figura 1. Para cada história foram elaboradas notas explicativas destinadas aos familiares e cuidadores que aprofundam as temáticas retratadas nas histórias, e questões norteadoras para as crianças. No Quadro 1, são apresentados o título, o tema e a sinopse de cada história.

**Figura 1:** Personagem “Morceguinho” nas versões colorida e em preto e branco

**Quadro 1:** Títulos das histórias infantis, temas retratados e sinopses

Títulos da história	Temas	Sinopses
A Princesa Coelha e o grito	Comportamento vocal abusivo, enfocando o grito	Apresentação do Reino dos Coelhos, da Princesa Coelha que se comunicava apenas por gritos, e de como a Fada fez com que ela entendesse que gritar prejudicava sua relação com seus interlocutores e provocava mudanças em sua voz.
O Pombo mensageiro	Alterações de leitura e escrita	Um filhote de pombo apresentava dificuldades na linguagem oral e escrita. Seu sonho era ser um mensageiro real igual a seus pais, mas não poderia devido às dificuldades. A Fada interviu, ensinando-o a falar e escrever bem, dessa forma conseguiu realizar seu sonho.
A Orelha boa	Perda auditiva unilateral e importância da reabilitação auditiva.	A Fada observava um filhote de morcego com dificuldade em escutar de uma orelha e na habilidade de localização sonora, o que impossibilitava seu voo. Reconhecendo a presença de uma perda auditiva, a Rainha Coelha encomendou um Aparelho de Amplificação Sonora Individual (AASI). O filhote se adaptou ao aparelho e conseguiu voar graças à intervenção.
O Sistema de Saúde	Implantação de um Sistema de Saúde gratuito e universal.	Foi criado o sistema de saúde pública pelos regentes do Reino dos Coelhos, e houve a apresentação pública da Fada. Foi esclarecido que os problemas poderiam ser solucionados sem o uso de mágica.
O Pato nadador	Otite do nadador e consequências para fala e audição.	Um dos filhotes de pato apresentava dor de ouvido após nadar e alterações na fala em consequência de otites de repetição. Foi apresentada uma forma de prevenir a dor de ouvido, e com isso a linguagem pôde ser desenvolvida sem dificuldades.
A Castor e a Chupeta	Uso da chupeta e impacto para o desenvolvimento orofacial.	Foram retratadas alterações dentárias pelo uso da chupeta, as quais impediam que a filhote usasse os dentes para roer madeira, atividade indispensável aos castores.
Chico e a Alimentação	Importância da variedade de alimentos para desenvolvimento do sistema estomatognático	Foi retratada a importância da variação alimentar, diferentes consistências, durezas, e tipos de alimento, para o desenvolvimento e fortalecimento do sistema estomatognático.
Lulu e o nariz entupido	Respirador oral e impacto no desenvolvimento orofacial e corporal.	Foi retratado o indivíduo respirador oral devido a um quadro alérgico. O objetivo foi mostrar mudanças anatômicas, das funções estomatognáticas, emocionais e cognitivas que esse tipo de respiração pode causar no desenvolvimento infantil.
Kiki e a Gagueira	Gagueira do desenvolvimento e *bullying*.	Um filhote de maritaca chamado Eduardo recebeu o apelido de Kiki, e sofria *bullying* na escola por apresentar gagueira desenvolvimental. No ambiente familiar não era muito diferente. Mesmo assim, se sentia motivado para atingir seu objetivo, ser repórter, como o pai.
A profissão da Fada	Papel do fonoaudiólogo. Importância da criação da Ciência Fonoaudiologia	A atuação da Fada ficou conhecida em outros reinos, e durante um evento para confraternização no Reino dos Coelhos, foi discutida a nomeação da profissão da Fada; e o nome escolhido foi Fonoaudiologia.

### Amostra: convite e acesso aos materiais

Os juízes foram convidados por meio das redes sociais das pesquisadoras. O convite disponibilizado instruía sobre o TCLE e sobre o acesso ao website que continha os materiais educativos.

O website foi criado exclusivamente para o fim de divulgação da pesquisa, para o grupo de juízes acessar os materiais, e era composto pelas seguintes páginas: Início (que descrevia a proposta do estudo); Histórias Infantis (com ilustrações e questões norteadoras); Notas Explicativas; Quem somos (com informações sobre os pesquisadores, a ilustradora e formas de entrar em contato). Havia, também, um destaque para o questionário *online* que deveria ser respondido pelos juízes do estudo após leitura e conferência de todo o website.

Para participar os juízes deveriam atender aos seguintes critérios de inclusão: ser fonoaudiólogo proveniente de serviços públicos e/ou privados, com tempo mínimo de atuação de dois anos, independentemente da área de atuação; ser educador da educação infantil e/ou primeiro ciclo do ensino fundamental de escolas públicas e/ou privadas, com tempo mínimo de atuação de cinco anos; ser familiar de crianças entre quatro e 10 anos. O gênero e unidade federativa dos juízes não foram considerados como critérios de inclusão. Na 1ª fase de validação, ocorrida entre março e maio de 2021, participaram 42 juízes: 10 fonoaudiólogos, 18 educadores e 14 familiares. Na 2ª fase, entre agosto e outubro do mesmo ano, 28 responderam ao novo questionário, sendo nove fonoaudiólogos, nove educadores e 10 familiares. A definição do número amostral por grupos seguiu recomendação de Lynn^(13)^, segundo o qual, um mínimo de seis juízes já assegura a validação do conteúdo analisado. Os indivíduos excluídos da amostra foram aqueles que aceitaram participar, mas não responderam ao questionário.

### Instrumento de coleta

O questionário continha duas partes: a primeira investigava dados pessoais e/ou profissionais dos juízes (cinco questões) e a segunda indagava sobre os materiais elaborados: conteúdo, vocabulário, ilustração, estrutura e motivação (20 questões). As questões que compunham os domínios investigados podem ser visualizadas no Quadro 2.

**Quadro 2:** Domínios e suas questões equivalentes sobre os materiais avaliados pelos juízes na 1ª fase de validação

Domínios	Nº	Questões
Conteúdo	Q1	O conteúdo das histórias está adequado ao público infantil.
	Q2	Foram apresentadas informações educativas relevantes ao público alvo.
	Q3	A sequência das histórias infantis é lógica e coerente.
	Q4	As histórias são importantes para o autocuidado de crianças.
	Q5	As histórias podem circular no meio científico da área.
	Q6	As informações contidas nas histórias podem ser consideradas como uma estratégia de educação em saúde.
	Q7	As questões norteadoras da compreensão do conteúdo para as crianças são coerentes.
	Q8	Os títulos das histórias são pertinentes.
	Q9	As notas explicativas para os familiares, educadores ou cuidadores são importantes.
	Q10	As notas explicativas para os familiares, educadores ou cuidadores são claras.
Vocabulário	Q11	O vocabulário é acessível ao público infantil.
	Q12	Os textos (das histórias e notas explicativas) são claros e objetivos.
Ilustração	Q13	As imagens são facilmente reconhecidas pelas crianças.
	Q14	As imagens despertam o interesse das crianças pelas histórias.
Estrutura	Q15	A formatação do texto, tamanho e fonte de letra é adequada.
	Q16	A organização do texto e ilustrações é adequada.
Motivação	Q17	O conteúdo é motivador para mudanças de comportamento.
	Q18	O conteúdo desperta interesse.
	Q19	Os materiais podem ser usados por qualquer cuidador.
	Q20	Os materiais propõem a construção de conhecimento para crianças e seus cuidadores.

Os juízes deveriam responder com base numa escala do tipo *Likert* de concordância de cinco pontos, que variava de 1 a 5, sendo 1= discordo totalmente; 2= discordo parcialmente; 3= não concordo nem discordo; 4= concordo parcialmente; 5= concordo totalmente, conforme recomendação da literatura recente^(7,12)^. Os juízes também podiam escolher a opção “não sei ou não quero responder”.

Para todas as questões, havia um espaço em branco para que o juiz comentasse sobre os itens, de modo a oferecer sugestões para a melhoria dos materiais. O tempo previsto para preenchimento do questionário foi de 30 minutos.

O *guideline Checklist for Reporting Results of Internet E-Surveys* (CHERRIES)^(14)^ foi utilizado por ser recomendado em pesquisas eletrônicas que envolvem questionários *online* para minimizar possíveis erros.

### Análise dos dados

Os dados foram organizados em planilha do Microsoft® Excel®, 2016. Para a análise descritiva das variáveis quantitativas foi calculada média, mediana e desvio padrão. As variáveis categóricas foram descritas por frequência e porcentagem.

A validade do conteúdo foi avaliada por medidas quantitativas e qualitativas. A análise qualitativa baseou-se nos comentários feitos pelos juízes. Em relação à análise quantitativa, foram calculados o Índice de Validade do Conteúdo (IVC) e o Coeficiente de Validade de Conteúdo (CVC), para verificar o grau de concordância dos juízes.

O IVC foi calculado a partir da divisão do número de juízes que concordaram parcial e totalmente com cada item investigado pelo número total de juízes. O cálculo foi feito para cada item, para cada domínio e, considerando cada grupo de juízes separadamente e geral (todos os juízes). A decisão por utilizar concordância total e parcial foi baseada no estudo de Alexandre e Coluci^(15)^. O índice mínimo de concordância entre os juízes sobre a decisão de alterar ou não as histórias, bem como as notas explicativas e as questões norteadoras foi de 80%, conforme recomendações de Lynn^(13)^ e Yusoff ^(16)^.

O CVC foi calculado de acordo com as respostas dos juízes, em cinco etapas:

1. Calculou-se a média de resposta de cada item investigado; 2) dividiu-se cada média pelo valor máximo possível de cada resposta (cinco), resultando em CVCi; 3) calculou-se o erro (Pei), para descontar possíveis vieses dos juízes para cada questão; 4) subtraiu-se o CVCi do Pei, resultando no CVCc de cada questão; 5) calculou-se o CVC total (CVCt) a partir da média de todas as questões para cada um dos domínios. Valores inferiores a 0,8 indicavam necessidade de revisão^(17)^.

## RESULTADOS

Em relação à caracterização da amostra, no que se refere aos fonoaudiólogos, a idade variou de 24 a 45 anos (média 33,2 anos, mediana 31 anos, e desvio padrão 8,7 anos). O tempo de atuação variou de dois a 20 anos (média 9,8 anos, mediana 7 anos, e desvio padrão 7,7 anos). A maioria atuava em linguagem (90%) e motricidade orofacial (60%). Os demais tinham experiência em outras áreas como fluência (40%), fonoaudiologia educacional (30%), audiologia (20%), voz (20%) e disfagia (20%).

Os educadores que participaram como juízes tinham idades entre 27 anos e 58 anos (média 43,1 anos, mediana 43 anos, desvio padrão 8,3 anos). O tempo de atuação profissional variou entre cinco e 33 anos (média 16,5 anos, mediana 16,5 anos, desvio padrão 7,3 anos). Em relação ao nível de ensino, 61% atuavam na educação infantil, 50% no ensino fundamental e 10% em centros de educação infantil.

Quanto aos familiares, a idade variou entre 24 anos e 51 (média 37,3 anos, mediana 36,5 anos, desvio padrão 8,1 anos). No que diz respeito ao nível de instrução, 43% concluíram ensino superior, 29% o ensino médio, 14% ensino fundamental, enquanto 14% não completaram o fundamental.

O IVC (por grupos de juízes e geral) para cada um dos domínios investigados (conteúdo, vocabulário, ilustrações, estrutura, motivação) da primeira fase pode ser visualizado na Tabela 1.

**Tabela 1:** Índice de Validade do Conteúdo (por categorias de juízes e geral) em relação ao domínio “conteúdo” e “vocabulário” dos materiais elaborados

Domínios	Resposta dos juízes em escala Likert de 5 pontos e cálculo de IVC (%)									
	Fonoaudiólogos (10)			Educadores (18)			Familiares (14)			Geral
CONTEÚDO	1, 2 ou 3	4 ou 5	IVC	1,2 ou 3	4 ou 5	IVC	1,2 ou 3	4 ou 5	IVC	IVC
O conteúdo das histórias está adequado ao público infantil.	0	10	100	0	18	100	0	14	100	100
Foram apresentadas informações educativas relevantes ao público.	0	10	100	0	18	100	0	14	100	100
A sequência das histórias infantis é lógica e coerente.	0	10	100	0	18	100	0	14	100	100
As histórias são importantes para o autocuidado de crianças.	0	10	100	0	18	100	0	14	100	100
As histórias podem circular no meio científico da área.	0	10	100	0	18	100	0	14	100	100
As informações contidas nas histórias podem ser consideradas como uma estratégia de educação em saúde.	0	10	100	0	18	100	0	14	100	100
As questões norteadoras da compreensão do conteúdo para as crianças são coerentes.	0	10	100	0	18	100	0	14	100	100
Os títulos das histórias são pertinentes.	0	10	100	0	18	100	3	11	**78,5** ^*^	92
As notas explicativas para os familiares, educadores ou cuidadores são importantes.	0	10	100	0	18	100	0	13	92	97,33
As notas explicativas para os familiares, educadores ou cuidadores são claras.	0	10	100	0	18	100	0	13	92	100
Total do Domínio			100			100			96	98,93
VOCABULÁRIO	1, 2 ou 3	4 ou 5	IVC	1,2 ou 3	4 ou 5	IVC	1,2 ou 3	4 ou 5	IVC	IVC
O vocabulário é acessível ao público infantil.	1	9	90	0	18	100	2	12	85	91,66
Os textos (das histórias e notas explicativas) são claros e objetivos.	1	9	90	0	18	100	0	14	100	96,66
Total do Domínio			90			100			92,5	94
ILUSTRAÇÃO	1, 2 ou 3	4 ou 5	IVC	1,2 ou 3	4 ou 5	IVC	1,2 ou 3	4 ou 5	IVC	IVC
As imagens são facilmente reconhecidas pelas crianças.	0	10	100	0	18	100	0	14	100	100
As imagens despertam o interesse das crianças pelas histórias.	0	10	100	1	17	94	1	13	92	95,33
Total do Domínio			100			97			96	97,66
ESTRUTURA	1, 2 ou 3	4 ou 5	IVC	1,2 ou 3	4 ou 5	IVC	1,2 ou 3	4 ou 5	IVC	IVC
A formatação do texto, tamanho e fonte de letra é adequada.	1	9	90	1	17	94	0	13	92	92
A organização do texto e ilustrações é adequada.	1	9	90	1	17	94	1	13	92	92
Total do Domínio			90			94			92	92
MOTIVAÇÃO	1, 2 ou 3	4 ou 5	IVC	1,2 ou 3	4 ou 5	IVC	1,2 ou 3	4 ou 5	IVC	IVC
O conteúdo é motivador para mudanças de comportamento.	0	10	100	0	18	100	0	13	92	97,33
O conteúdo desperta interesse.	1	9	90	0	18	100	0	14	100	96,66
Os materiais podem ser usados por qualquer cuidador.	1	9	90	0	18	100	1	13	92	92
Os materiais propõem a construção de conhecimento para crianças e seus cuidadores.	0	10	100	0	18	100	0	14	100	100
Total do Domínio			95			100			96	97
IVC Geral			96			98,2			94,5	95,92

* Valor inferior ao mínimo de concordância de 80%

Muitos comentários positivos foram feitos pelos juízes: “As histórias contextualizam o universo lúdico das crianças”; “A sequência das histórias dá a impressão de continuidade, como se o reino fosse real”; “Muito instrutivo e cativante”, “Ajudam a trabalhar e entender as situações cotidianas da profissão”; “Meu filho de sete anos concluiu sozinho o aprendizado ao final de cada história”.

Apenas o aspecto “título” do domínio “conteúdo” obteve IVC inferior a 80% para o grupo de familiares, enquanto o CVC foi limítrofe em 0,81. O comentário feito por um juiz fonoaudiólogo auxiliou na compreensão do baixo IVC, permitindo modificações nos títulos de três histórias:

*“Todos os títulos abordam a personagem principal, com exceção da história do morcego (‘A Orelha boa’). Na história ‘A princesa coelha e o grito’ eu mudaria a estrutura da frase para “O grito da princesa”, e também não achei o título ‘Sistema de saúde’ interessante/ convidativo para o público infantil”.*

Apesar de todos os aspectos do domínio “vocabulário” ter apresentado IVC acima de 80%, as pesquisadoras fizeram modificações em algumas palavras da história a partir dos comentários feitos por dois juízes (familiar e fonoaudiólogo, respectivamente): “dar um exemplo sobre o que é *bullying*”, “dique é uma palavra de difícil entendimento a depender da idade”.

Em relação aos domínios “ilustração” e “estrutura”, o IVC foi superior a 80% e os comentários dos juízes foram positivos: “formatação suficiente”, “está nítido”, “organização perfeita”.

Apesar dos IVC para o domínio “Motivação” ter sido acima de 80%, um comentário feito pelo grupo de familiares despertou a atenção das pesquisadoras para mudanças necessárias: “talvez para crianças maiores, de nove ou 10 anos, o conteúdo não seja suficiente para motivá-las pelo fato de ser bastante infantil”, “achei que as histórias estão um pouco extensas, dependendo da idade”.

Os valores de CVC de cada item investigado variaram de 0,81 a 1,0. Já para o CVC total dos domínios, de acordo com os grupos de juízes (Figura 2), os valores variaram de 0,91 a 0,98, conforme apresentado a seguir: para o “conteúdo”, o CVCt dos educadores foi de 0,93, familiares 0,96, e fonoaudiólogos 0,98, assim respectivamente para os domínios restantes “vocabulário”, 0,92, 0,95 e 0,91; “ilustração”, 0,92, 0,96 e 0,93; “estrutura”, 0,92, 0,94 e 0,91; e “motivação”, 0,92, 0,97, e 0,94. O CVC geral foi de: familiares, 0,96; educadores, 0,92; e fonoaudiólogos, 0,94.

**Figura 2:** Valores de CVCt dos domínios investigados para cada categoria de juiz na primeira fase de validação

Após a análise qualitativa dos juízes houveram alterações em três principais aspectos: 1) título das histórias, sendo que o título “A Princesa Coelha e o grito” foi substituído por “O grito da Princesa Coelha”, “A Orelha boa” por “A Orelha do Morcego” e “O Sistema de Saúde” por “Saúde para todos”; 2) vocabulário: o termo *bullying* foi explicado e a palavra “dique” foi substituída por “barragem”; 3) idade mínima recomendada: foi aumentada de quatro para cinco anos.

Na segunda fase de validação, após as alterações realizadas de acordo com as análises qualitativas e quantitativas, os juízes responderam ao novo questionário composto por: “O novo vocabulário é acessível ao público infantil” e “Os novos títulos das histórias são pertinentes”. O IVC pode ser visualizado na Tabela 2.

**Tabela 2:** Índice de Validade do Conteúdo da 2ª fase de validação dos materiais elaborados

Questões	Resposta dos juízes em escala Likert de 5 pontos e cálculo de IVC (%)									
	Fonoaudiólogos (9)			Educadores (9)			Familiares (10)			Geral
	1, 2 ou 3	4 ou 5	IVC	1,2 ou 3	4 ou 5	IVC	1,2 ou 3	4 ou 5	IVC	IVC
O novo vocabulário é acessível ao público infantil	0	9	100	1	8	88,88	2	8	80	89,62
Os novos títulos das histórias são pertinentes.	0	9	100	1	8	88,88	0	10	100	96,29
Total			100			88,88			90	92,95

O CVC de vocabulário e título para os familiares foi de 0,97 e 0,96; respectivamente; educadores, 0,88 e 0,9; e fonoaudiólogos, 0,97 em ambos.

## DISCUSSÃO

O processo de validação dos materiais educativos envolveu diferentes olhares sob o mesmo objeto^(7)^, bem como diferentes análises para seu aperfeiçoamento.

Em relação aos familiares, a maior parte apresentou nível de instrução entre o ensino médio completo e o fundamental incompleto, o que também tem sido referido pelo censo brasileiro. Segundo o Instituto Brasileiro de Geografia e Estatística (2020), em pessoas com 25 anos ou mais, a minoria apresenta ensino superior completo (17,4%)^(18)^.

A diversidade de nível de instrução dos familiares foi favorável, pois permitiu que os materiais fossem julgados por pessoas de distintas vivências e cenários sociais. Não houve relatos sobre dificuldade de compreensão do conteúdo das histórias pelo vocabulário utilizado, ou do entendimento da proposta dos materiais, o que sugere que eles podem ser utilizados por familiares independentemente do nível de instrução.

Da mesma forma, a variedade de fonoaudiólogos quanto às especialidades e de educadores quanto aos níveis de ensino foi importante para o julgamento dos materiais.

Os resultados quantitativos, de acordo com o IVC e CVC da primeira fase, indicaram alto valor de aceitabilidade, apontando apenas necessidade de revisão nos títulos das histórias. A análise qualitativa, feita a partir dos comentários dos juízes, revelou outros aspectos a serem melhorados, o que indica a importância de integrar os dois tipos de análises.

Em relação aos cinco domínios analisados, o “conteúdo”, o “vocabulário” e a “motivação” foram os que receberam sugestões de melhoria pelos juízes. De acordo com a literatura^(19)^, o conteúdo e o vocabulário são os domínios que mais sofrem modificações na validação de materiais educativos.

Modificar vocabulário implica excluir, acrescentar e/ou substituir palavras e reformular frases^(19)^. No presente estudo, houve adequação do vocabulário ao público infantil nas histórias, visando a facilitação da compreensão do conteúdo pelo leitor^(20)^. Embora a palavra “dique” pudesse favorecer a expansão do vocabulário por ser uma palavra não corriqueira, a mudança para “barragem” objetivou facilitar a explicação e entendimento sobre o processo de construção realizado pelos castores, personagens de uma das histórias. A exemplificação de comportamentos atrelados ao *bullying*, como rir e apelidar, também foi importante para ampliar o entendimento da criança sobre esse tipo de violência.

No domínio “conteúdo”, no que se refere ao teor das histórias, houve boa aceitação pelos juízes, havendo, apenas, mudanças no título de três histórias.

As questões norteadoras para a compreensão do conteúdo foram consideradas coerentes pelos juízes. Contar histórias com a mediação de questões norteadoras permite a checagem do conteúdo apreendido, além de aumentar a complexidade do discurso narrativo^(21)^.

Em relação ao domínio “motivação”, os materiais elaborados foram considerados capazes de despertar interesse, gerar conhecimentos em crianças e cuidadores, e mudar comportamentos. No entanto, conforme sugestões feitas pelos juízes, considerou-se importante modificar a faixa etária do público alvo das histórias. Aumentou-se a idade mínima para cinco anos, e será recomendado que até sete anos a contação seja intermediada por adultos, conforme recomendação do Ministério da Saúde^(22)^. Crianças maiores de sete anos, que já tenham adquirido código escrito da língua portuguesa, devem ser estimuladas a realizar leituras sozinhas para que seja potencializada sua autonomia e tomada de decisões^(23)^.

As ilustrações das histórias infantis foram bem aceitas pelos juízes. O uso desse recurso visual capta a atenção do leitor^(6,24)^, podendo enfocar alguns elementos específicos que contribuem para o aprendizado^(24)^.

As alterações realizadas possibilitaram o aumento da qualidade dos materiais elaborados^(25)^, além de evidenciarem o empenho de cada juiz contribuinte^(26)^. A especificidade dos apontamentos dos juízes facilitou o processo de reescrita dos materiais. A segunda fase de validação, feita após as modificações, também indicou valores acima do mínimo de concordância de 80% para IVC e CVC. Os itens “vocabulário” e “títulos” foram novamente apreciados e bem aceitos pelos juízes.

A importância dos materiais foi percebida pelos juízes, de acordo com os resultados quantitativos e análise qualitativa. Esses juízes reconheceram que as histórias infantis são boas estratégias de educação em saúde, podendo favorecer o desenvolvimento do autocuidado infantil^(26)^, além de divulgar a importância da atuação fonoaudiológica. Fonoaudiólogos, familiares e profissionais da educação poderão utilizar essa ferramenta para discutir assuntos sobre a saúde infantil com os educandos e seus cuidadores.

As limitações do estudo se relacionaram ao decréscimo do número de juízes entre a primeira e a segunda fase de validação dos materiais, de 42 para 28 juízes. Embora existente, essa diminuição não influenciou o número mínimo de juízes necessários para o processo de validação. Uma das possíveis causas pode ter sido o intervalo de tempo entre as fases de validação.

Espera-se que os materiais elaborados e validados sejam publicados em formato de livro, digital e físico, e divulgados para fonoaudiólogos, educadores e familiares de crianças visando a educação em saúde e o estímulo à leitura.

## CONCLUSÃO

Os materiais elaborados tiveram boa aceitabilidade pelo grupo de juízes. O processo de validação reforçou a importância de combinar diferentes pessoas e análises no aperfeiçoamento de ferramentas educativas.

Os comentários dos juízes foram fundamentais para apontar as mudanças necessárias: três títulos foram modificados, termos técnicos foram explicados e/ou substituídos, e a idade mínima sugerida para acesso aos materiais foi aumentada para cinco anos.

Após as modificações realizadas, espera-se que os materiais possam ser utilizados na rotina do profissional da Fonoaudiologia e da Educação, e possam despertar o interesse de crianças e familiares sobre a Fonoaudiologia no desenvolvimento infantil, favorecendo reflexões sobre o autocuidado na infância.
